# Human Eosinophils Express the High Affinity IgE Receptor, FcεRI, in Bullous Pemphigoid

**DOI:** 10.1371/journal.pone.0107725

**Published:** 2014-09-25

**Authors:** Kelly N. Messingham, Heather M. Holahan, Alexandra S. Frydman, Colleen Fullenkamp, Rupasree Srikantha, Janet A. Fairley

**Affiliations:** 1 Department of Dermatology, University of Iowa, Iowa City, Iowa, United States of America; 2 Veterans Administration Medical Center, Iowa City, Iowa, United States of America; The Ohio State University, United States of America

## Abstract

Bullous pemphigoid (BP) is an autoimmune blistering disease mediated by autoantibodies targeting BP180 (type XVII collagen). Patient sera and tissues typically have IgG and IgE autoantibodies and elevated eosinophil numbers. Although the pathogenicity of the IgE autoantibodies is established in BP, their contribution to the disease process is not well understood. Our aims were two-fold: 1) To establish the clinical relationships between total and BP180-specific IgE, eosinophilia and other markers of disease activity; and 2) To determine if eosinophils from BP patients express the high affinity IgE receptor, FcεRI, as a potential mechanism of action for IgE in BP. Our analysis of 48 untreated BP patients revealed a correlation between BP180 IgG and both BP180 IgE and peripheral eosinophil count. Additionally, we established a correlation between total IgE concentration and both BP180 IgE levels and eosinophil count. When only sera from patients (n = 16) with total IgE≥400 IU/ml were analyzed, BP180 IgG levels correlated with disease severity, BP230 IgG, total circulating IgE and BP180 IgE. Finally, peripheral eosinophil count correlated more strongly with levels of BP180 IgE then with BP180 IgG. Next, eosinophil FcεRI expression was investigated in the blood and skin using several methods. Peripheral eosinophils from BP patients expressed mRNA for all three chains (α, β and γ) of the FcεRI. Surface expression of the FcεRIα was confirmed on both peripheral and tissue eosinophils from most BP patients by immunostaining. Furthermore, using a proximity ligation assay, interaction of the α- and β-chains of the FcεRI was observed in some biopsy specimens, suggesting tissue expression of the trimeric receptor form in some patients. These studies provide clinical support for the relevance of IgE in BP disease and provide one mechanism of action of these antibodies, via binding to the FcεRI on eosinophils.

## Introduction

Bullous pemphigoid (BP) is an autoimmune skin disease resulting in antibody-mediated separation of the epidermis from the dermis. The initial phase of lesion development is characterized by urticarial plaques and eosinophilic infiltration of the upper dermis. As the lesions progress, formation of tense, fluid-filled vesicles corresponds histologically to loss of epidermal adhesion at the basement membrane zone (BMZ). In addition, there is perilesional infiltration of lymphocytes, mast cells and neutrophils, and often, elevated levels of circulating IgE [1], [2].

The severity of BP is correlated with levels of autoantibodies targeting the hemidesmosomal protein BP180, also known as type XVII collagen [3]–[6]. These autoantibodies are comprised primarily of the IgG and IgE classes, and predominantly target the non-collagenous 16A (NC16A) region of the BP180 protein [7]–[11]. Although most studies have focused on the pathogenicity of IgG-class autoantibodies in BP, the contribution of IgE autoantibodies has also been demonstrated *in vitro* and *in vivo* [10], [12]–[14]. Most importantly, a critical role for IgE was established through successful treatment of steroid-unresponsive BP patients with omalizumab, a humanized monoclonal antibody which binds IgE, thus inhibiting receptor interaction [13], [15], [16]. Despite these advances, the mechanism(s) of IgE-mediated autoimmunity in BP, and many other autoimmune diseases, are not well understood.

There is increasing evidence that eosinophils are powerful mediators of immunity through the production of a variety of cytokines, toxic granule proteins, and lipid mediators [17], [18]. Eosinophils have been shown to play a role in a multitude of inflammatory and/or autoimmune diseases; including, asthma and allergy, a growing number of eosinophil-associated gastrointestinal diseases and other hypereosinophilic syndromes [17], [19], [20]. A role for eosinophils in BP pathogenesis is supported by the presence of degranulated eosinophils and extracellular eosinophil granules or free granule proteins within the lesion [21]–[25]. These observations were extended by elegant studies examining serial skin biopsies of developing lesions, which illustrated that eosinophil degranulation precedes blister formation [26]. These findings led to the hypothesis that eosinophil infiltration is a contributor to lesion formation, rather than a consequence of tissue damage. The fact that tissue eosinophilia is only seen in models of BP that utilize IgE [12], [14] indicates that these two factors may be related.

In humans, the high-affinity IgE receptor, FcεRI, is comprised of an α-chain, which is responsible for IgE binding, and β- and γ-chains, which mediate signal transduction [27]. The receptor is present in either tetrameric (αβγ_2_) or trimeric (αγ_2_) forms, and is expressed on a variety of cells, including mast cells, basophils and eosinophils [27], [28]. In diseases characterized by high IgE and eosinophilia, mRNA for the FcεRI chains has been detected in circulating eosinophils [29]–[33]. Surface expression of the FcεRI has also been described [30], [33], [34]; however, levels are often low and are complicated by the presence of intracellular stores of the receptor [29], [35]. Due to the extremely high affinity (K_a_∼10^10^ M^−1^) of FcεRI for IgE, antibody is always bound to receptors expressed on the cell surface [36], so some studies examine bound IgE an index of receptor expression [37]. Interpretation of these studies is difficult because eosinophils also express the low affinity IgE receptor, CD23/FcεRII [38]. Although eosinophil expression of FcεRI α-chain was reported on a limited number of BP biopsies using a “mirror section” staining method [39], these studies utilized a single-step fixation/permeabilization and did not examine peripheral eosinophils or establish which receptor form, tetrameric or trimeric, was present.

Clinical studies indicate a critical role for IgE autoantibodies in BP; however, the exact nature of its contribution is not well understood. As eosinophils are increasingly recognized as mediators of autoimmunity, establishing relationships between eosinophilia and other markers of disease severity may provide additional insight into the mechanisms of the disease process in BP. In this study, we characterized a large cohort of untreated BP patients and examined associations between circulating IgE, eosinophil numbers, IgG and IgE autoantibody levels and disease severity. Furthermore, FcεRI expression was evaluated by mRNA expression, flow cytometry and two immunofluorescent (IF) staining methods to demonstrate that tissue specific expression of this receptor may impact eosinophil function in BP.

## Materials and Methods

### Patients and samples

Serum samples were collected from 48 untreated patients (23 females, 25 males, mean age 78.2 years, range 59–97) diagnosed with BP based on clinical (cutaneous blistering, erythematous/urticarial plaques), histologic (subepidermal blistering on biopsy), and direct IF (linear IgG and/or C3 deposition at the basement membrane zone) criteria. Patients were enrolled at the time of diagnosis and had received no prior treatment for BP. Since these samples were collected over several years, disease severity was assessed using the previously reported BP Index [40] scaled from 1–6 (remission to severe disease), or in combination with the recently instituted BP Disease Area Index (BPDAI) criteria [41]. Using the BPDAI, skin disease is scored from 0–120 (least to most severe). None of our patients had mucosal involvement. Four patients were not scored for disease severity upon enrollment since a diagnosis of BP had not been confirmed. Control sera were obtained from age- and gender-matched patients with no known autoimmune disease (N = 58; 25 female, 33 male, mean age 79.9 years, range 63–98) or dermatology patients with other autoimmune diagnoses (N = 25; 16 female, 9 male, mean age 75.9, range 48–93), including atopy, eczema, pemphigus vulgaris and urticarial vasculitis.

De-identified archival blocks of lesional skin biopsies from BP patients were obtained from the University of Iowa Department of Pathology. Skin, discarded at the time of cutaneous surgery, was collected from age- and gender-matched controls with no known autoimmune disease. This study was approved by the Institutional Review Board (IRB #200701758) at the University of Iowa and was performed in adherence to the Declaration of Helsinki Guidelines. Written informed consent was obtained prior to inclusion in the study. The Institutional Review Board waived the need for informed consent to obtain 1) de-identified archival blocks of lesional skin biopsies from BP patients; and 2) skin discarded at the time of surgery.

### Eosinophil enrichment, flow cytometry and cytospin preparation

Eosinophils were enriched from peripheral blood of BP or control subjects using density gradient centrifugation and immunoselection. Briefly, blood was diluted 1∶2 in PBS, subjected to density gradient centrifugation (Ficoll-Paque, Mediatech Inc.) and granulocytes were obtained after hypotonic lysis of the RBCs. Eosinophils were purified by immunomagnetic negative selection according to kit instructions (Miltenyi Biotec). For flow cytometry, cells were stained on ice with mixtures of CD16 (Alexa-Flour 488 or APC), CD49d-PE, CD203c-PerCP Cy5.5 (Biolegend, San Diego, CA) and FcεRIα-APC (R & D Systems), or FcεRIβ (Abnova) followed by goat anti mouse IgG Alexa Fluor 488 (Molecular Probes) or anti human IgE-FITC (Bethyl Laboratories). CD203c staining was used eliminate basophils. This purification routinely results in >93% purity of CD203c^−^/CD49d^+^/CD16^−^ eosinophils. Cytospins were prepared using freshly purified cells (4×10^4^) on glass microscope slides with a Shandon 2 cytospin. Eosinophil purity was also confirmed using Wright’s stain (Fisher Scientific).

### RNA Isolation, Reverse Transcription and PCR

RNA was extracted using an RNeasy Plus mini kit and followed by a genomic DNA Eliminator spin column (Qiagen). The RNA was collected using RNase- free water and quantified using NanoDrop. The RNA was DNase treated using the TURBO DNA-free ™ Kit as directed (Ambion, Life Technologies). One step RT-PCR was performed as follows: RT at 55°C for 30 minutes and inactivation at 94°C for 2 minutes; 35 cycles of PCR utilized 94°C denaturation, 15 sec, 57°C annealing, 30 sec, and 68°C extension for 1 minute. Final extension was at 68°C for 7 minutes. PCR products were run on a 1.5% agarose gel. PCR Primers: Beta Actin F5′-GGA CTT CGA GCA AGA GAT GG-3′ and Beta Actin R5′-AGC ACT GTG TTG GCG TAC AG-3′; IgE Alpha F5′-TGT GGC AGC TGG ACT ATG AG-3′ and IgE Alpha R5′-GAA ATG TGA CCT GCT GCT GA-3′; IgE Beta F5′-TAT TGA AGT CGG CCT CAT CC-3′ and IgE Beta R5′-TCC CCA GAA TGG ATA ACC TG-3′; IgE Gamma F5′-GGA GAG CCT CAG CTC TGC TA-3′ and IgE Gamma R5′- CAG GCA TAT GTG ATG CCA AC-3′.

### Immunofluorescent staining and proximity ligation assay

For IF, cytospins or biopsy cryosections (5 µM) were fixed in 50% methanol: acetone for 5 min, washed in tris-buffered saline (TBS, pH 7.2) and stained in TBS/5% BSA. For MBP staining, cells were permeabilized with 5% Triton x100 in TBS. Primary antibodies included those specific for the following: MBP (clone BMK13, EMD Millipore), FcεRIα (clone 9E1, Abcam), human IgE (polyclonal, Antibodies Online) or appropriate isotype controls. Species specific secondary antibodies conjugated to Alexa-546 or Alexa-488 (Molecular Probes) were used. Nuclei were counter-stained with diamidino-2-phenylindole (DAPI). Staining was visualized using a Nikon photomicroscope equipped with epifluorescence. Images were pseudo-colorized and occasionally adjusted for overall brightness using NIH Image J (National Institutes of Health).

The proximity ligation assay (PLA) [42] was conducted as described by the manufacturer (*In situ* PLA, OLINK Bioscience). Briefly, slides were fixed in 4% paraformaldehyde, washed, and incubated with primary antibodies specific for human IgE (goat polyclonal, Invitrogen), FcεRIα (clone 9E1, Abcam,) and/or FcεRIβ (goat polyclonal, Santa Cruz Biotechnology). The proximity of bound antibodies was evaluated using species specific probes. If probes bind to sample in close proximity (<40 nm) to each other, ligation and amplification of the signal occurs. Images were captured using a Zeiss 710 confocal microscope at the University of Iowa Central Microscopy Research Facility and processed with NIH Image J (National Institutes of Health).

### ELISA

Commercially available ELISA kits were used to evaluate the following: BP180 and BP230 IgG, EDN (MBL International, Japan). BP180-specific IgE was quantified using a previously described protocol [40]. Total IgE levels were quantitated using electrochemiluminescence performed by the pathology laboratory services at the University of Iowa.

### Degranulation assay

The degranulation assay was adapted from studies evaluating mast cell degranulation [2]. Briefly, peripheral blood was obtained from BP patients or controls (which included healthy controls (n = 11) and those with other autoimmune skin diseases (n = 3; atopy, pemphigus, psoriasis)). Whole blood was exposed to the recombinant NC16A domain of BP180 (10 µg/ml) expressed as a glutathione S-transferase (GST) fusion protein [43] or an equimolar concentration of GST protein for 30 min at 37°C. Samples were also treated with 100 µg/ml ionomycin (maximal non-immunologic release), buffer alone (spontaneous release), or 0.5% Triton X-100 (total release). In some cases, the NC1 domain of type XVII collagen (a generous gift from Mei Chen, University of Southern California) was used as an additional control (not shown). Cell-free supernatants were collected and assayed for EDN. The average GST value was subtracted from the average NC16A value for each duplicate sample. The percent total release was calculated as follows: ((NC16A−spontaneous)/(ionomycin−spontaneous))×100.

### Statistics

Experiments were conducted with the number of individual patient samples indicated as N and results were expressed as mean ± SD. Assays utilizing human cells or tissues were conducted with duplicate or triplicate samples from the same patient being averaged and represented as n = 1. Statistical analysis was performed using GraphPad Prism software, version 5.0 (GraphPad Software, San Diego, CA). A non-parametric unpaired T-test (Mann-Whitney U-test) or ANOVA (Kruskal-Wallis) was used to determine statistical significance between groups. Spearman’s rank correlation coefficient (r) was used to determine the statistical dependence between variables. The P-value of ≤0.05 was considered to be statistically significant.

## Results

### Clinical and immunologic characterization of study subjects

**Figure 1:**
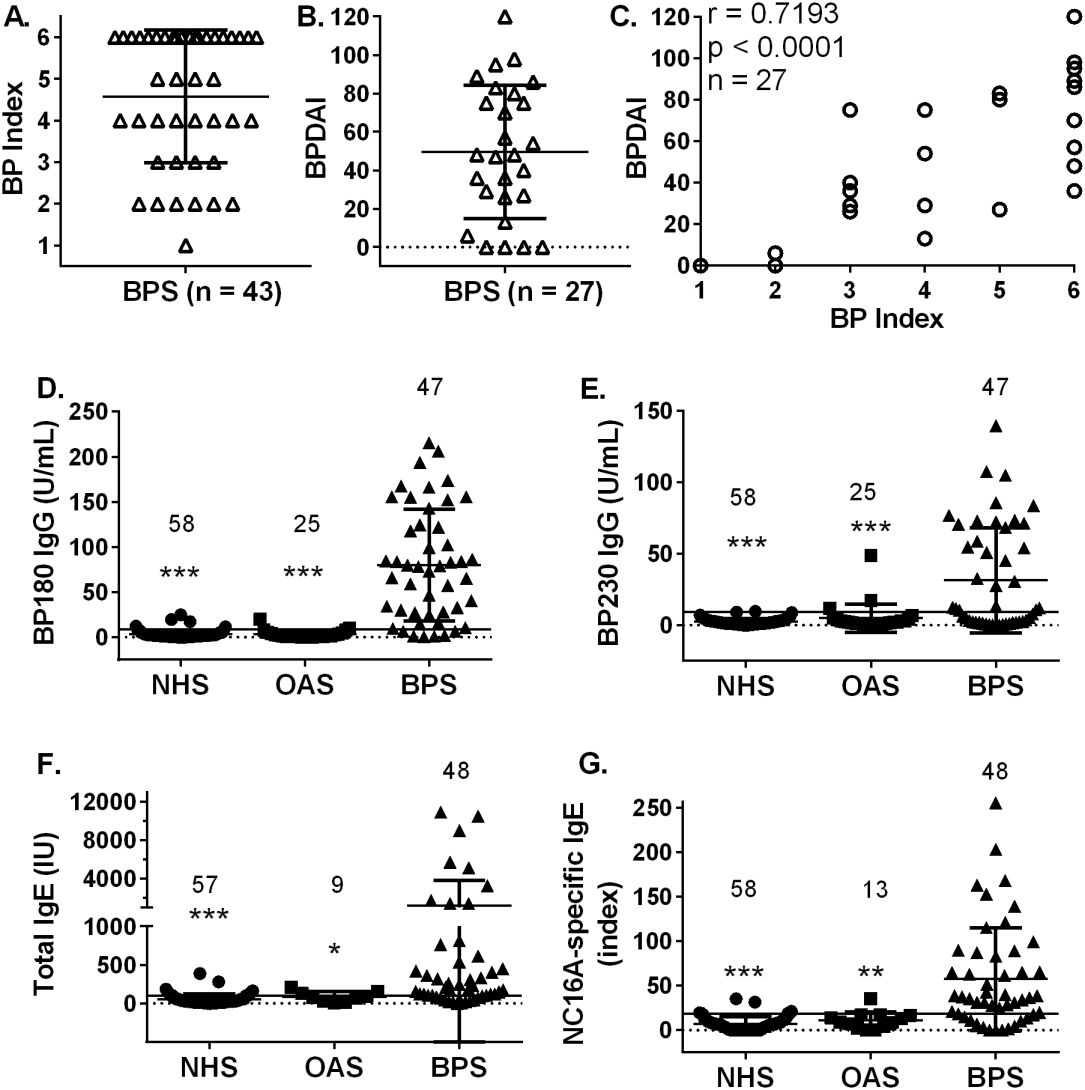
Disease activity and autoantibody profiles in patients with BP. Subjects (n = 48) were enrolled before receiving any immunosuppressive treatment for BP. Since patients were enrolled over several years, disease activity was scored with a BP Index (A) or the more comprehensive Bullous Disease Area Index (BPDAI) criteria (B). A strong correlation (Spearman’s r = 0.7193, p<0.0001) was observed between these two scoring systems (C). Sera were collected from untreated BP patients (BP sera; BPS), patients evaluated for other autoimmune skin diseases (other autoimmune sera; OAS), or age- and gender-matched controls (normal human sera; NHS) and evaluated for BP180 IgG, BP230 IgG, total IgE and BP180 IgE by ELISA. Each point represents the average of duplicate samples from an individual patient with the N per group indicated. Mann-Whitney U-test, *p≤0.05, **p≤0.01, ***p≤0.001.

Patients with a confirmed diagnosis of BP who had not received any prior immunosuppressive treatment were enrolled in the study. Disease activity was assessed using either a BP Index [40] alone, or in combination with the recently established BPDAI criteria [41] (Figure 1A, B). In the patients (n = 27) scored using both systems, a high degree of correlation was observed between BP index and BPDAI scores (Figure 1C, Spearman’s r = 0.7193, p<0.001). Patients evaluated with the BPDAI had an average score of 49.56±34.59 with an average number of days between reported onset of disease and confirmed diagnosis was 103.58±79.78.

Sera were collected from untreated BP patients (BP sera; BPS), patients evaluated for other autoimmune skin diseases (other autoimmune sera; OAS), or age- and gender-matched controls (normal human sera; NHS) and evaluated for BP180 IgG, BP230 IgG, total IgE and BP180 IgE by ELISA (Figure 1 D–G). BP patients with IgG reactivity to regions of BP180 outside the immunodominant NC16A region [44] were not included in this study. Circulating eosinophil counts were also obtained for most BP patients. As expected, BPS exhibited significantly elevated levels of BP180 IgG and BP230 IgG when compared to either OAS or NHS. In addition, both total IgE and BP180-specific IgE were also significantly increased in BPS compared to either control group. Furthermore, elevated levels of *both* BP180 specific IgG and IgE were detected 75% (36/48) of BP samples, which is similar to our previous findings [40]. Circulating eosinophil counts from BP patients (mean 694.4/mm^3^±1110, range 10–5830) were also above the normal range (40–390/mm^3^).

To better understand the role of IgE and eosinophils in BP, we evaluated these factors in relation to the BP180 and BP230 IgG levels and disease activity. A correlation matrix was performed so that relationships between all variables could be explored. The strength of these associations is expressed as Spearman’s correlation coefficient (r) with the likelihood this relationship is due to chance decreasing as the p value gets smaller (Table 1). As expected in BP, a strong correlation between BP180 IgG and disease severity is observed. In addition, BP180 IgG also correlates with BP180 IgE levels and circulating eosinophil count. Total IgE concentration correlates with BP 230 IgG and, to a lesser extent, BP180 IgE levels and eosinophil count. No correlations were observed with age or gender of study subjects.

**Table 1:** Correlation^a^ between antibody levels, eosinophil counts, and disease severity in untreated BP patients.

	BP180 IgG	BP230 IgG	Total IgE^b^	BP180 IgE^c^	BP Index/BPDAI^d^
**BP180 IgG**	–	0.268	0.203	0.370*	0.734***/0.653***
**BP230 IgG**	0.268	–	0.429**	0.218	0.231/0.201
**Total IgE**	0.203	0.429**	–	0.331*	0.235/0.117
**BP180 IgE**	0.370*	0.218	0.331*	–	0.364/0.317
**Eos count**	0.430**	0.347*	0.273*	0.251	0.364/0.393*

a determined using Spearman’s rank correlation coefficient (r) and p = *<0.05, **<0.01, ***0.001.

b reported as IU/ml, normal range ≤100.

c expressed as Index Units, positive test ≥19.

d reported as BP index/BPDAI as described in the Methods.

When analysis was focused on sera from patients (n = 16) with levels of total IgE≥400 IU/ml, some additional relationships were revealed (Table II). BP180 IgG levels correlated strongly with disease severity, but also with BP230 IgG, total circulating IgE and BP180 IgE. The concentration of total IgE was additionally correlated with disease severity and BP230 IgG levels. Although total IgE and BP180 IgE increased together in most BP samples (r = 0.438), this was not statistically significant. Interestingly, peripheral eosinophil count correlated more strongly with levels of BP180 IgE then with BP180 IgG in this cohort. Overall, these analyses provide additional evidence for a relationship between IgE autoantibodies and peripheral eosinophilia in BP.

**Table 2:** Correlation^a^ of autoantibody level, eosinophil counts and disease activity^b^ in untreated BP patients with total IgE>400 IU (n = 16).

	BP180 IgG	BP230 IgG	Total IgE^c^	BP180 IgE^d^	BP Index/BPDAI^e^
**BP180 IgG**	–	0.559*	0.512*	0.738**	0.782**/0.667*
**BP230 IgG**	0.559*	–	0.656**	0.309	0.582*/0.01
**Total IgE**	0.512*	0.656**	–	0.438	0.414**/0.681
**BP180 IgE**	0.738**	0.309	0.438	–	0.577*/0.739
**Eos count**	0.535*	0.433	0.449	0.642*	0.462/0.595

a determined using Spearman’s rank correlation coefficient (r) and p = *<0.05, **<0.01, ***0.001.

b disease activity was evaluated using one or both methods (BP index/BPDAI) as described in Methods.

c reported as IU/ml, normal range ≤100.

d expressed as Index Units, positive test ≥19.

e reported as BP index/BPDAI as described in the Methods.

**Figure 2:**
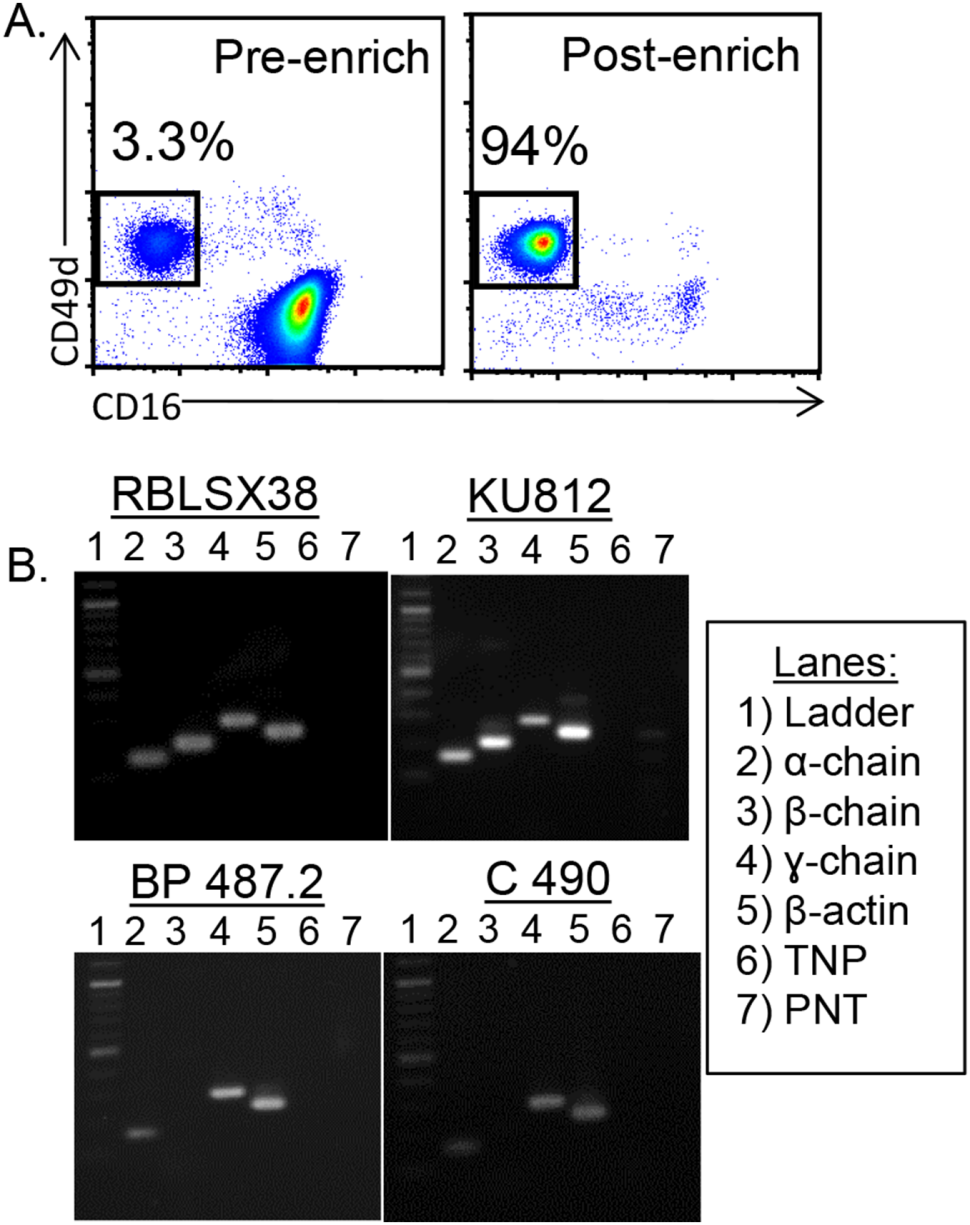
Purified peripheral eosinophils express FcεRI mRNA. Peripheral eosinophils were purified via immunomagnetic negative selection to >94% purity (A) and RT-PCR was performed (B). Conditions were optimized using a rat basophil line (RBLSX38) expressing all 3 chains of the human FcεRI. The myeloid cell line, KU812, expressed mRNA for all 3 chains of the receptor, while examination of most BP patients and controls revealed only α- and γ-chain mRNA. Additional control lanes included TNP (template, no primer) and PNT (primer, no template). Representative images are shown; all samples are described in Table III.

To investigate the possibility of a direct link between IgE and modulation of eosinophil activity in BP, we utilized RT-PCR to detect mRNA of the FcεRI α-, β- and γ-chain [29], [45], [46] in peripheral eosinophils from BP patients (n = 6), healthy controls (n = 3) or patients evaluated for non-BP diseases associated with eosinophilia and/or high IgE (n = 4). Eosinophils were immunomagnetically purified (>94%) from peripheral blood to eliminate basophils (Figure 2A). BP samples were obtained from patients with eosinophil counts ranging from 10–950 cells/mm^3^ and circulating IgE ranging from 34–3855 IU/ml. Two control cell lines were utilized: RBL-SX-38, a rat basophilic line which expresses all three chains of the human FcεRI (a gift of Dr. Jean P. Kinet, Harvard Medical School [47]; and KU812, a human line established from a myeloid leukemia [48] (Figure 2B). As shown in Table III, samples from 4/6 BP patients, 3/3 healthy controls and 2/4 controls with other autoimmune diseases co-expressed α-and γ-chain mRNA. In contrast, only 1/6 BP samples, and none of the controls, expressed β-chain mRNA (Table III). Interestingly, no receptor mRNA was detected in 2/6 BP and 2/4 other autoimmune control samples, despite a positive result with the β-actin primers in the same sample/reaction. Moreover, receptor chain expression did not coincide with concentration of circulating IgE (total or BP180 specific) or peripheral eosinophilia in these samples.

**Table 3:** Patient characteristics and mRNA expression of FcεRI α, β, and γ receptor chains^a^.

	α-chain	β-chain	γ-chain	TotalIgE^b^	NC16AIgE^c^	eosinophilcount	diseaseactivity^d^
*healthy controls*							
C493	+	–	+	142	4.9	ND^e^	–
C498	+	–	+	7	18.0	ND	–
C499	+	–	+	24	ND	ND	–
*Bullous pemphigoid*							
BP323	+	–	+	<1	0	200	2/ND
BP422	–	–	–	3855	93.4	950	6/67
BP487	+	–	+	115	6.2	ND	3/30
BP501	+	+	+	34	89.2	ND	6/48
BP502	+	–	+	289	4.8	350	4/18
BP504	–	–	–	90	9.4	260	6/58
*Other autoimmune*							
C503: urticarial vasculitis	+	–	+	770	0	250	5/49
C508: scabies	–	–	–	571	0	10	5/ND
C497: eczematous dermatitis	+	–	+	1842	3.0	750	5/25
C488: papulopustular eczema	–	–	–	64	71.2	710	5/79

a mRNA expression was evaluated by RT-PCR after immunomagnetic purification of eosinophils.

b reported as IU/ml, normal range ≤100.

c expressed as Index Units, positive test ≥19.

d reported as BP index/BPDAI as described in the Methods.

e Not done.

**Figure 3:**
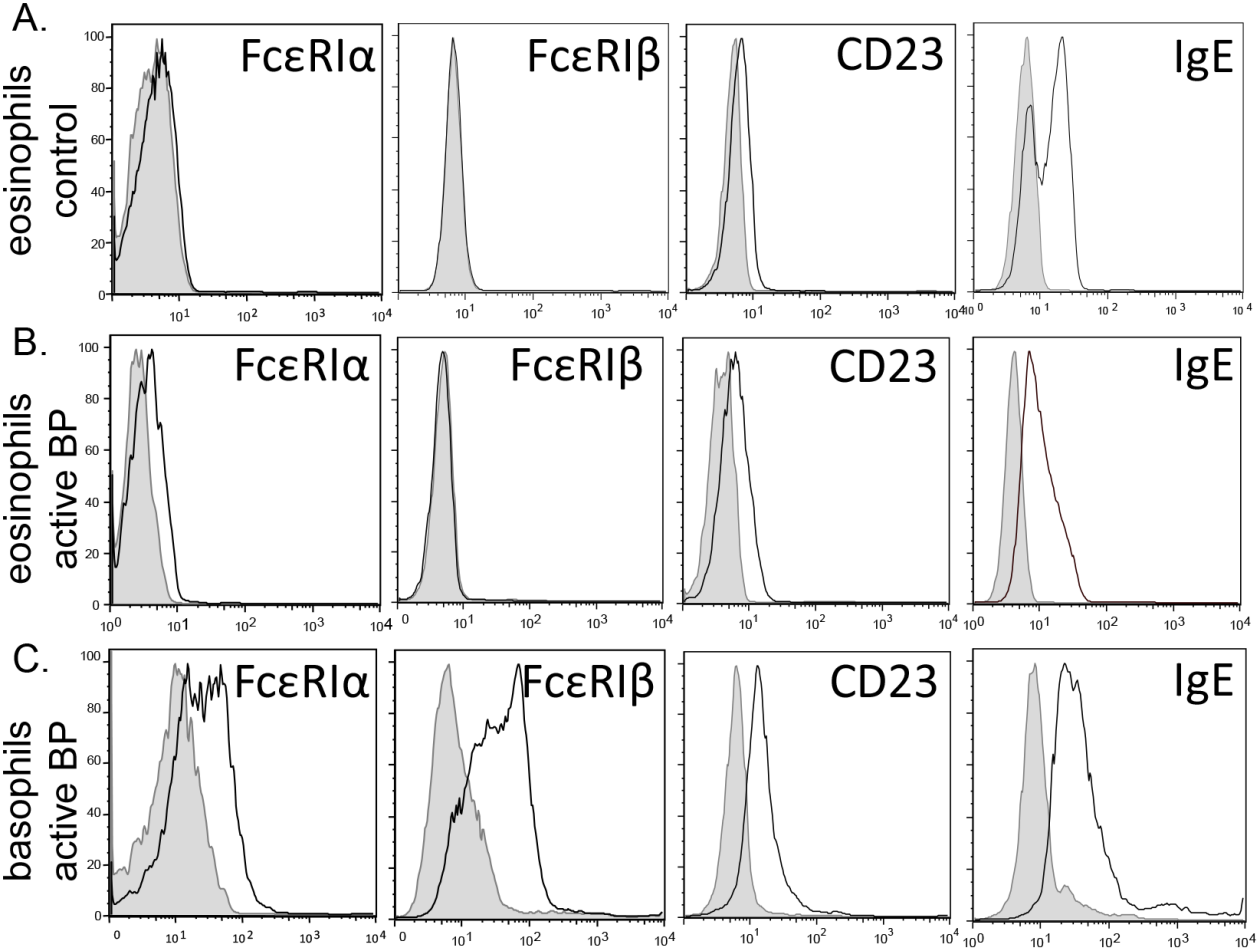
Evaluation of IgE receptor expression on circulating eosinophils from BP patients. Peripheral granulocytes were stained with fluorescently tagged antibodies specific for human CD203c, CD49d, CD16, and FcεRI-α, FcεRIβ, CD23 or IgE for flow cytometric analysis. Eosinophils were identified by gating on the CD16^−^/CD49d^+^/CD203c^−^ population. Eosinophils from healthy controls (A), active BP patients (B), or basophils (CD16^−^/CD49d^+^/CD203c^+^) from active BP patients (C) were evaluated. The degree of specific staining is indicated by the open histogram compared to appropriate isotype control shown by the shaded histogram. Staining is representative of 10 BP patients and 11 age- and gender-matched controls.

To evaluate surface expression of FcεRI on peripheral eosinophils from BP patients, four color flow cytometry was employed using antibodies specific for FcεRI-α or –β on peripheral eosinophils (CD203c^−^/CD16^−^/CD49d^+^). Peripheral blood granulocytes were enriched via density centrifugation prior to staining. FcεRI was not detected on eosinophils from controls subjects, regardless of circulating IgE concentration (n = 11 with low IgE, n = 7 with IgE≥400) (Figure 3A). Eosinophils obtained from 7/10 untreated patients with active BP exhibited a low level of surface staining for FcεRI α-chain (depicted in representative histograms, Figure 3B), but not the β-chain, while the remaining active patients did not differ from controls. Furthermore, eosinophil FcεRI expression was not detected on any samples from BP patients in remission (n = 6; not shown). Since peripheral basophils are known to express the tetrameric FcεRI, receptor expression was examined on the CD203c^+^/CD16^+/dim^/CD49d^+^ population. Robust expression of both the α- and β-chains was observed on basophils from active BP patients (Figure 3C), or controls (not shown).

Eosinophils were also examined for surface expression of CD23 and bound IgE. As expected, CD23 was detected on eosinophils from 10/10 BP patients and controls (Figure 3A, B). Likewise, basophils expressed higher levels of CD23 than eosinophils isolated from the same patient (Figure 3C). Within BP patients, CD23 expression was variable and did not correspond with circulating IgE levels. Surface bound IgE was also evaluated since this has previously been used as a surrogate for functional IgE receptor expression [37]. As expected based on the receptor staining, a similar level of IgE staining was observed on eosinophils from BP patients (active or remission) and controls, which was increased on basophils (Figure 3). Furthermore, removal of in vivo bound IgE with lactic acid did not increase detection of FcεRI α- or β-chains (data not shown), or CD23, thus eliminating the possibility that bound antibody blocked receptor detection.

**Figure 4:**
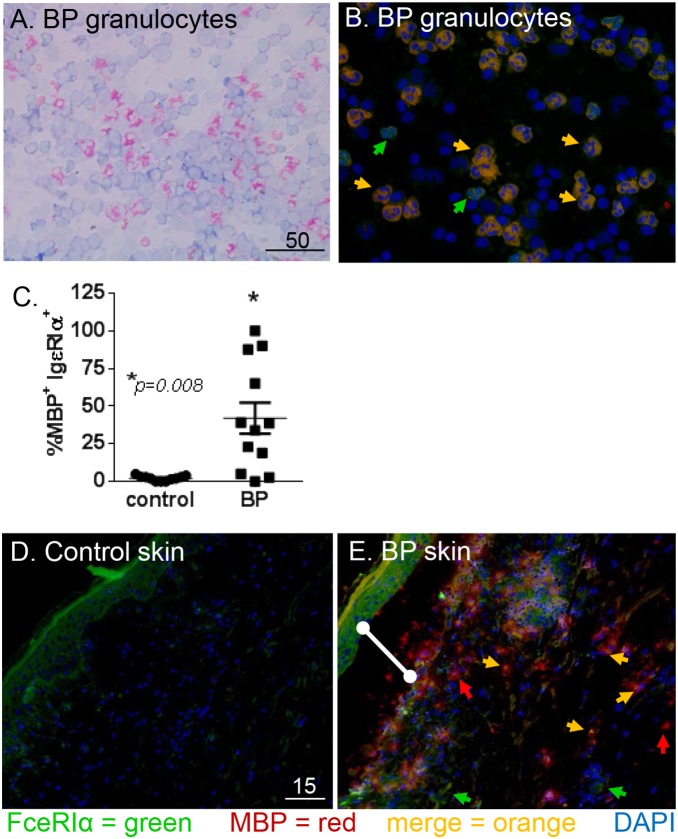
Eosinophils in BP biopsies and circulation express FcεRI. Peripheral granulocytes were adhered to glass slides and Wright’s stained (A) or stained with antibodies directed against FcεRIα (green), MBP (red) and DAPI nuclear stain (blue). Expression of FcεRIα by BP eosinophils is indicated by co-localization on merged image (orange arrowheads) (B). Cumulative data indicating the frequency of circulating MBP^+^ cells also expressing FcεRIα in BP patients (n = 12) or controls (n = 11). Mann-Whitney test (*P* = 0.008) (C). To evaluate receptor expression within the lesion, cryosections (5 uM) of control (D) or lesional skin (E) were stained as above. The BP skin shows a subepidermal split (E, white bar) and numerous infiltrating eosinophils (red), some of which express the FcεRIα (orange arrows). Eosinophils lacking FcεRIα expression (red arrow) and other non-MBP^+^ cells that are FcεRIα^+^ (green arrows) are also visible. Staining is representative of 9 BP and 7 control samples. Scale bar is represented in µM.

Identification of tissue eosinophils is usually achieved by antibody staining of intracellular granule proteins, such as major basic protein (MBP). Immunofluorescent staining was utilized to evaluate expression of the FcεRI α-chain (green) on MBP^+^ eosinophils (red) from the circulation (n = 12) and lesional skin (n = 9) of BP patients (Figure 4). Normal blood (n = 11) and skin (n = 7) was obtained from controls, but the frequency of eosinophils in these samples is very low (Figure 4C). Staining of peripheral eosinophils from patients with active BP revealed a high degree of variability of FcεRIα expression, with the proportion of dual-positive cells ranging from 0 to 100%. The frequency of eosinophils from healthy controls that expressed FcεRIα was ≤5%. A Mann-Whitney test revealed a significantly (*P* = 0.008) higher frequency of circulating FcεRIα^+^/MBP^+^ cells in 12 BP patients, compared to 11 controls. As expected in the BP skin [21], [22], many MBP^+^ cells were observed in both the blister cavity (indicated by the bar) and the dermis of biopsied skin (Figure 4B, D). A subset of the MBP^+^ eosinophils co-expressed the FcεRI as indicated by the orange arrows. Additionally, FcεRI expression was also noted on cells that did not stain with MBP antibodies (Figure 4B, D, green arrows), which were likely basophils based on their size, nuclear morphology and prevalence in BP skin. Due to the paucity of MBP^+^ eosinophils in normal skin, statistical comparison was not possible.

Thus far, our RT-PCR, flow cytometry and IF staining suggest that eosinophils from BP patients express the FcεRI; however, the level of surface staining does not provide enough resolution to determine which receptor form, tetrameric (αβγ^2^) or trimeric (αγ^2^), is expressed. To address this, we employed an *in situ* PLA to assess interaction of high affinity receptor chains and provide signal amplification. Antibodies specific for IgE and the α- and β-chains of FcεRI were used to label slides of enriched peripheral eosinophils or matched biopsy tissue from the same patient. The γ-chain of the FcεRI was not examined as it is present in both receptor forms [28]. High resolution confocal microscopy was used to confirm eosinophilic lineage via the unique nuclear morphology of PLA positive cells.

**Figure 5:**
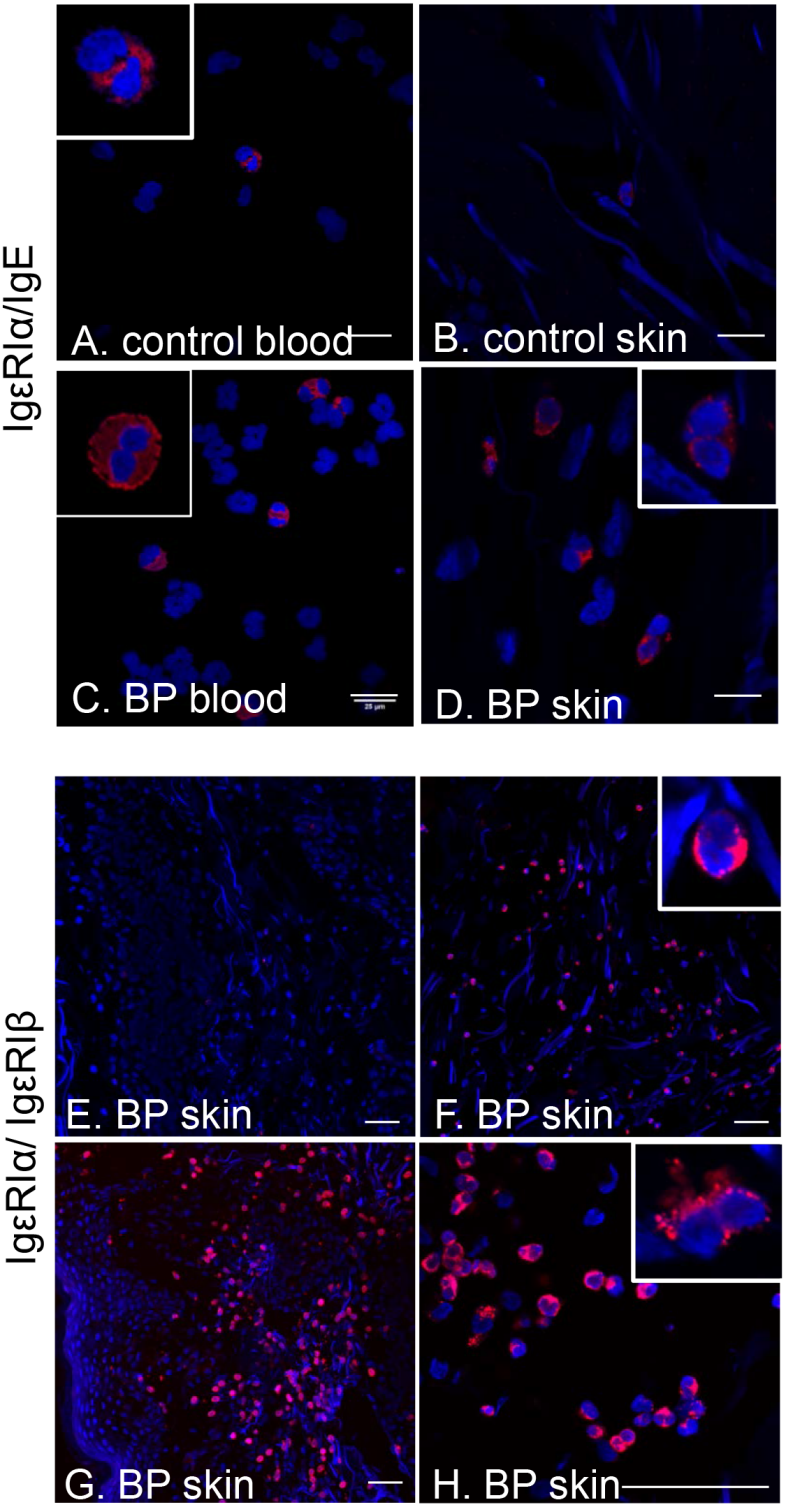
Surface expression of FcεRI on BP eosinophils. Interaction of FcεRIα with IgE or FcεRIβ was evaluated using the proximity ligation assay on non-permeabilized preparations of circulating granulocytes or skin cryosections from BP patients or controls. Eosinophils were identified by their unique nuclear morphology (bi-lobed nucleus stained with DAPI) using high resolution confocal microscopy. Interaction of FcεRIα/IgE on peripheral blood and tissue eosinophils from BP patient (A, B) or controls (C, D). Insets are enlarged to show nuclear morphology. Scale bar = 25 uM. Interaction of FcεRIα/FcεRIβ on eosinophils in lesional biopsies (E–H). Panel H is an image of the same BP sample depicted in panel G, captured at higher magnification for resolution of nuclear morphology. Scale bar = 50 uM.

Since circulating IgE is normally bound to FcεRI expressed on the cell surface, we first examined the co localization of these two molecules using the PLA to confirm extracellular receptor expression. In this assay, red fluorescence is observed only if both molecules are located within close proximity (≤40 nm) to one another, indicating interaction. Eosinophils were identified by their characteristic bi-lobed nucleus (see insets, Figure 5). Interestingly, co-expression of FcεRI and IgE was occasionally observed on enriched peripheral eosinophils from controls, and within normal skin (Figure 5A, B), although eosinophils are rare in these samples. In confirmation of our flow cytometry and IF studies, there is clear co-expression of FcεRI and IgE on the surface of eosinophils from the blood and biopsy tissue of BP patients (Figure 5C & D, Movie S1). These studies confirm surface expression of the FcεRI by eosinophils. To further characterize this receptor expression, we utilized the PLA to evaluate colocalization of the FcεRI α- and β-chains on slides containing peripheral eosinophils or matched skin biopsies from the same patients (n = 5, Table IV). This approach allows for examination of tissue specific differences in FcεRI expression. Co-expression of the α- and β-chains was not evident on slides of circulating eosinophils from BP patients (Table IV), nor was it observed on control samples (n = 4, circulating cells or skin, data not shown). In contrast, co-expression of the FcεRI α- and β-chains was observed in BP skin, indicating tissue expression of the tetrameric receptor form (Figure 5F–H, Table IV, Movie S2). Expression of the tetrameric receptor form varied greatly from sample to sample as evidenced by the four individual biopsy samples shown (Figure 5E–H, Table IV). These studies confirm eosinophil FcεRI expression in BP and indicate that the tetrameric form of the receptor is up-regulated upon migration to the lesion site.

**Table 4:** Samples evaluated for FcεRI expression using the proximity ligation assay.

	*peripheral blood* a		*lesional biopsy*					
	FcεRI/IgE	FcεRIα/FcεRIβ	FcεRIα/IgE	FcεRIα/FcεRIβ	totalIgE^b^	BP180-IgE^c^	eosinophilcount	diseaseactivity^d^
BP456	+	–	+++	+++	538	30.78	130	0/2
BP470	+	–	+	+	408	152.6	720	3/75
BP474	–	–	+	+	34	115.2	490	5/83
BP 482	++	–	+++	–	124	99.2	530	3/40
BP485	–	–	–	–	166	15.4	ND^e^	4/12

a eosinophils were enriched from peripheral blood using density centrifugation and adhered to glass slides.

b expressed as IU of IgE, normal range ≤100 IU.

c ELISA Index Value, normal range ≤19.

d reported as BP index/BPDAI as described in the Methods.

e not done.

**Figure 6:**
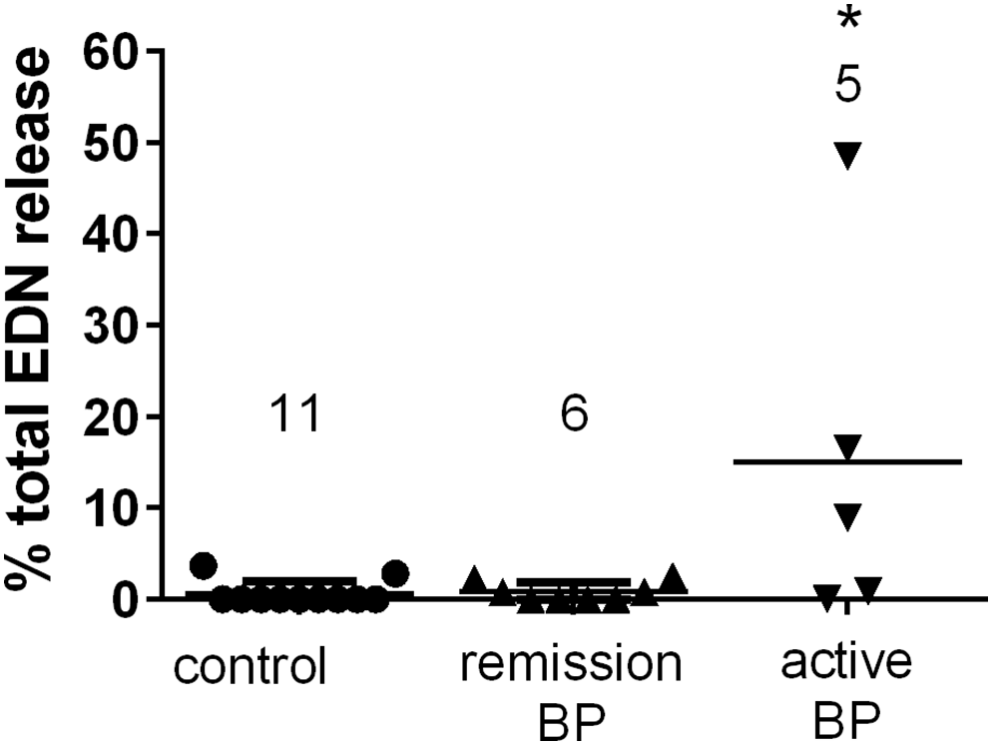
Eosinophils from active BP patients degranulate in response to BP180 protein. For degranulation, peripheral blood was incubated with 10 µg/ml NC16A or GST control protein in duplicate and EDN release was measured by ELISA. Mean GST values (background) were subtracted from NC16A and results are expressed as percent maximal (100 nM ionomycin) release. Each point represents the mean of replicate samples from the same patients. The number of patients per group is indicated. A Kruskal-Wallis test was performed, * = p≤0.05.

The presence of BP180-specific IgE, coupled with eosinophil FcεRI expression, provides a potential mechanism for antigen specific responses in BP. Eosinophils primarily respond to FcεRI engagement through release of preformed mediators via piecemeal degranulation [17], [49]. Thus, the ability of peripheral eosinophils to degranulate in response to BP180 was determined using an ex vivo assay (Figure 6) [2]. Peripheral eosinophils were enriched from flaring (untreated) BP patients, BP patients in remission (no disease activity, without immunosuppressive medications for 12–35 months) or aged healthy controls. Equal numbers of cells were exposed to the recombinant GST fusion protein encoding the immunodominant NC16A domain of BP180 (10 ng/ml) or GST alone for 30 min *in vitro*. Cell free supernatants were collected and assayed for the presence of the eosinophil derived neurotoxin (EDN). Antigen specific degranulation is indicated as a percentage of total (non-immunologic) release triggered by ionomycin (100 µg/ml) for each sample. Antigen-specific EDN release was observed only in a subset (3/5) of samples from active BP patients, while no BP180-specific degranulation was observed in eosinophils from patients in remission (n = 8) or controls (n = 13). Additional studies will elucidate the mechanisms of eosinophil degranulation and its relevance to BP.

## Discussion

BP patients often exhibit increased serum IgE levels accompanied by peripheral or lesional eosinophilia. Previously, we and others proposed a relationship between circulating total IgE [1], [37], [50], or BP180 specific IgE [37], [40], [51], [52], and the severity of clinical BP. Additionally, there is evidence for eosinophil degranulation in lesional tissue [22], [26] and, recently, we have shown that peripheral eosinophil numbers more accurately reflect disease activity than IgG autoantibody levels [53]. Despite these observations, the connection between IgE and eosinophils in BP is not firmly established.

The current studies reveal a significant correlation between eosinophil count and disease severity when a large number of untreated BP patients were evaluated. However, the strongest associations were observed in the subset of patients with circulating IgE≥400 IU/L where i) total IgE correlated strongly with disease severity; and ii) peripheral eosinophil counts correlated with both total and BP180 specific IgE. These studies provide further clinical evidence for a link between IgE and eosinophil activation in BP.

Eosinophil expression of FcεRI is reported in diseases associated with elevated circulating IgE and eosinophilia [33], [45], [46], [54]; however, receptor expression by eosinophils from healthy donors remains controversial [35], [55], [56]. In this report, we detected mRNA for the FcεRI α- and γ-chains in purified circulating eosinophils from both BP patients and healthy controls. These findings are consistent with the expression of the trimeric (αγ_2_) FcεRI, which is largely accepted as the receptor form expressed by eosinophils [33], [45], [54], [57]. Interestingly, we also report FcεRI β-chain mRNA in 1/6 BP samples Circulating eosinophils obtained from atopic patients [29] or those with hypereosinophilic syndromes [46] have also been reported to express β-chain mRNA. While this observation suggests BP eosinophils are capable of expressing the tetrameric (αβγ_2_) receptor form under certain conditions, mRNA expression does not necessarily reflect expression on the cell surface. Furthermore, the complete lack of receptor mRNA in some samples, despite elevated IgE and/or eosinophilia, further underscores the complex nature of eosinophil FcεRI receptor expression. In humans, IL-4 is required for the production of the FcεRI α-chain [58]. Thus, differences in cytokine milieu, which varies by individual, likely contribute to the disparities in FcεRI mRNA expression in our studies.

Typically, eosinophil surface expression of FcεRI is quite low when compared to basophils [56], and does not appear to correlate with IgE concentrations [20], [28], [35], [59]. To eliminate complications due to the presence of intracellular stores of FcεRI receptor chains, we utilized surface staining of FcεRI α-chain on CD203c^−^/CD16^−^/CD49d^+^ eosinophils. Surface FcεRI α-chain is representative of an intact receptor since the α-chain is not translocated to the surface in the absence of the γ-chain [28]. Detection of the γ-chain alone is not helpful, since it is shared by other receptors [28]. Similar to previous reports in parasitic infection, atopy or allergy [30], [33], [34], a low level FcεRI α-chain was detected, which was not improved by lactic acid stripping of IgE. In contrast, robust staining was observed when surface-bound IgE was evaluated by flow cytometry.

CD23 is known to be expressed on the surface of eosinophils and basophils where it plays a role in cellular activation and degranulation. In our hands, CD23 expression was detected on peripheral eosinophils from BP patients and controls as well as basophils from BP patients. Furthermore, interaction of CD23 with IgE was detected on the surface of a variety of cells in lesional biopsies and normal skin using the PLA (data not shown). Thus, evaluation of surface bound IgE likely represents total IgE receptor expression, rather than FcεRI or CD23 alone. Since the clinical manifestations of BP are exhibited in the skin, eosinophil FcεRI expression was evaluated in lesional biopsies using two approaches. In the first, eosinophils were identified by the presence of eosinophil major basic protein (MBP), which requires permeabilization of the samples. FcεRI staining prior to permeabilization resulted in loss of signal, therefore colocalization of FcεRI α-chain protein with MBP reflects both surface bound and intracellular receptor stores [29], [35]. Secondly, we utilized an in situ PLA on non-permeabilized samples to detect interaction of FcεRIα with IgE or FcεRIα with FcεRIβ. An additional benefit of this assay is that it provides several-hundred-fold signal amplification if target molecules interact [42]. While this assay confirmed FcεRI expression by BP eosinophils, examination of matched blood and biopsy samples from the same patients revealed several differences in FcεRI expression. The most remarkable finding is that lesional eosinophils may express the tetrameric (αβγ_2_) receptor form in some patients.

To our knowledge, this is the first report of the tetrameric (αβγ_2_) FcεRI on the surface of eosinophils in the skin; however, others [32], [33] have reported β-chain mRNA on eosinophils in skin from atopic donors. IgE receptor expression has not been characterized in other skin diseases known to have a prominent eosinophilic infiltrate, including, but not limited to, other immunobullous diseases, allergic contact dermatitis, drug eruption, arthropod assault, erythema neonatorum toxicum, Churg-Strass disease, angiolymphoid hyperplasia with eosinophilia, incontinentia pigmenti and Langerhans cell histiocytosis. Additional samples will need to be evaluated to determine if eosinophil expression of the tetrameric (αβγ_2_) receptor in BP is associated with IgE levels, eosinophilia, disease activity, or some other factor. It is well established that the antigenic crosslinking of the tetrameric FcεRI on basophils results in degranulation [28]; however, the role of IgE receptors in eosinophil degranulation is debated [18], [29], [30], [55]. Our in vitro studies demonstrate eosinophil degranulation in response to BP180 antigen in a subset of active patients. Interpretation of these studies is limited by the fact that samples were a mixed population of granulocytes and do not rule out the possibility that eosinophil degranulation could be mediated indirectly by factors released upon basophil degranulation. Indeed, we have previously shown via histamine release that BP basophils degranulate upon exposure to BP180 [2], and basophil derived mediators could also trigger eosinophil degranulation [60]. To investigate the possibility of indirect eosinophil degranulation, triggered by mast cells, degranulation of immunomagnetically purified (≥94%) eosinophils has been examined on a limited number (3 BP, 3 control) of samples. RT-PCR was conducted on the same samples to confirm receptor mRNA. Thus far, no degranulation has been observed despite detection of mRNA for all 3 receptor chains (data not shown). Studies continue to investigate whether patient-dependent variations in surface receptor expression or an indirect mechanism of degranulation might account for these findings.

In summary, these studies utilized clinical samples to explore the relationships between IgE autoantibodies and eosinophilia in BP. In patients with IgE≥400 IU/ml, peripheral eosinophil count correlated strongly with IgE autoantibodies directed against BP180. Expression of FcεRI by circulating and lesional eosinophils from BP patients provides a novel mechanism of action for IgE in BP. Furthermore, these studies suggest that expression of the tetrameric (αβγ_2_) FcεRI may contribute to eosinophil degranulation in BP lesions.

## Supporting Information

Movie S1 **Representative interaction of FcεRI and IgE on the surface of eosinophils in BP lesions.** Interaction of specific molecules was evaluated using the proximity ligation assay on non-permeabilized skin cryosections from BP patients. Eosinophils were identified by their unique bi-lobed nuclear morphology (DAPI stain, blue) on z-series image stacks using high resolution confocal microscopy. Interaction of FcεRIα/IgE on tissue eosinophils from BP patient results in a red fluorescent signal. The image stacks have been smoothed and pseudo-colored in NIH ImageJ. Scale bar = 50 uM.
(AVI)

Movie S2 **Representative interaction of FcεRIα and FcεRIβ on the surface of eosinophils in BP lesions.** Interaction of specific molecules was evaluated using the proximity ligation assay on non-permeabilized skin cryosections from BP patients. Eosinophils were identified by their unique bi-lobed nuclear morphology (DAPI stain, blue) on z-series image stacks using high resolution confocal microscopy. Interaction of FcεRIα/FcεRIβ on tissue eosinophils from BP patient results in a red fluorescent signal. Circled area contains several eosinophils. The image stacks have been smoothed, annotated and pseudo-colored in NIH ImageJ. Scale bar = 50 uM.
(AVI)
